# Electrochemical Performance and Hydrogen Storage of Ni–Pd–P–B Glassy Alloy

**DOI:** 10.3390/nano12234310

**Published:** 2022-12-05

**Authors:** Ahmed Alshahrie, Bassim Arkook, Wafaa Al-Ghamdi, Samah Eldera, Thuraya Alzaidi, Hassan Bamashmus, Elsayed Shalaan

**Affiliations:** 1 Physics Department, Faculty of Science, King Abdulaziz University, Jeddah 21589, Saudi Arabia; 2 Center of Nanotechnology, King Abdulaziz University, Jeddah 21589, Saudi Arabia; 3 Physics and Astronomy Department, University of California, Riverside, CA 92521, USA; 4 Physics Department, Faculty of Science, Albaha University, Albaha 65779, Saudi Arabia; 5 Physics Department, Faculty of Science, Al-Azhar University, Cairo 11751, Egypt; 6 College of Engineering, University of Business and Technology (UBT), Jeddah 23847, Saudi Arabia

**Keywords:** hydrogen storage, electrochemical dealloying, metallic glass, supercapacitors

## Abstract

The search for hydrogen storage materials is a challenging task. In this work, we tried to test metallic glass-based pseudocapacitive material for electrochemical hydrogen storage potential. An alloy ingot with an atomic composition of Ni_60_Pd_20_P_16_B_4_ was prepared via arc melting of extremely pure elements in an Ar environment. A ribbon sample with a width of 2 mm and a thickness of 20 mm was produced via melt spinning of the prepared ingot. Electrochemical dealloying of the ribbon sample was conducted in 1 M H_2_SO_4_ to prepare a nanoporous glassy alloy. The Brunauer–Emmett–Teller (BET) and Langmuir methods were implemented to obtain the total surface area of the nanoporous glassy alloy ribbon. The obtained values were 6.486 m^2^/g and 15.082 m^2^/g, respectively. The Dubinin–Astakhov (DA) method was used to calculate pore radius and pore volume; those values were 1.07 nm and 0.09 cm^3^/g, respectively. Cyclic voltammetry of the dealloyed samples revealed the pseudocapacitive nature of this alloy. Impedance of the dealloying sample was measured at different frequencies through use of electrochemical impedance spectroscopy (EIS). A Cole–Cole plot established a semicircle with a radius of ~6 Ω at higher frequency, indicating low interfacial charge-transfer resistance, and an almost vertical Warburg slope at lower frequency, indicating fast diffusion of ions to the electrode surface. Charge–discharge experiments were performed at different constant currents (75, 100, 125, 150, and 200 mA/g) under a cutoff potential of 2.25 V vs. Ag/AgCl electrode in a 1 M KOH solution. The calculated maximum storage capacity was 950 mAh/g. High-rate dischargeability (HRD) and capacity retention ($S_{n}$) for the dealloyed glassy alloy ribbon sample were evaluated. The calculated capacity retention rate at the 40th cycle was 97%, which reveals high stability.

## 1. Introduction

Metallic glass is a material with unique properties that make it attractive for use in various applications [1,2,3,4,5,6,7]. It is strong and lightweight, yet highly conductive. Metallic glass is typically made via rapid cooling of molten metals from above their critical temperature. This process traps the atoms in a disordered state, preventing them from forming the long-range atomic order characteristic of a crystalline structure and thus creating a non-crystalline structure [8,9,10]. This gives the material its strength, stiffness, and good corrosion resistance. The material also has excellent electrical and thermal conductivity. While metallic glass has many attractive properties, its main drawback is its brittleness. It is also difficult to work with, as it cannot be cast or forged as traditional metals can. Despite these challenges, metallic glass is being used in certain applications where its unique properties are advantageous. For example, it is being used to create strong, lightweight materials for use in aerospace and automotive applications [6]. It is also being used to create electrical and electronic devices with improved performance. Since most research on metallic glass has focused on its mechanical properties, corrosion resistance, and magnetic properties, metallic glass-based materials are not expected to be directly used in portable energy-storage devices.

Supercapacitors have recently attracted great interest as promising energy-storage devices due to their fast charging and discharging, high power density, and long lifetime, along with excellent cycle performance and outstanding stability [11,12,13,14]. Supercapacitors produce higher-power density compared to batteries and store more energy than capacitors store. Currently, porous carbon is used to manufacture supercapacitors due to its high surface area and good electrical conductivity. However, there is always a need to develop new porous materials that improve overall performance of the devices.

Therefore, supercapacitors made with nanoporous metallic glass can be charged and discharged very rapidly, making them ideal for use in applications where quick bursts of power are needed, such as electric vehicles, due to their corrosion-resistant properties. They can also store more energy than can traditional batteries, making them ideal for use in renewable energy systems where power needs to be stored for long periods of time [15,16].

Pseudocapacitors are devices that can store a large amount of energy in a small footprint. They are made up of two electrodes, one positive and one negative, separated by an electrolyte [17,18]. The electrode can be made from a variety of materials, including carbon (porous carbon, nanotubes), metal oxides, and conducting polymers [19,20]. When voltage is applied to the electrodes, ions in the electrolyte are attracted to the electrodes and create an electric field. This electric field stores energy in the form of an electric potential difference between the two electrodes. Pseudocapacitors have several advantages over traditional capacitors. They can be charged and discharged much more quickly than traditional capacitors and hold more charges. While supercapacitors rely entirely on physical storage of energy, pseudocapacitors include both physical and chemical storage. Pseudocapacitors are used in a variety of applications, including cell phones, laptops, and electric vehicles. They are also being researched for use in grid storage applications and as backup power supplies for medical devices [21].

Hydrogen is the lightest element on the periodic table and has potential to be used as a clean, renewable energy source. Hydrogen can be used in fuel cells to produce electricity with water as the only by-product. It can also be used in internal combustion engines and is being developed for use in aviation. However, one of the challenges of using hydrogen is storing it in a way that is safe and efficient [22]. Electrochemical hydrogen storage offers a promising solution to this problem [23,24].

The topic of hydrogen storage is important for several reasons. First and foremost, it is a technology that can enable widespread use of hydrogen as an energy carrier. Secondly, it is a critical enabling technology for fuel-cell systems, which convert chemical energy into electricity using hydrogen and oxygen. There are a few different methods of storing hydrogen, each with its own advantages and disadvantages [25]. The most common method is to store it in compressed gas cylinders. This method is well-suited for large-scale applications or factories. Another method is to store hydrogen in metal hydrides. Metal hydrides are the most promising candidate material for solid hydrogen storage [26]. Among them, Ni-based alloys have gained much attention for hydrogen storage and related applications [27]. Addition of Pd to the alloy increases storage capacity due to the catalytic effect of Pd. Hydrogen absorption and desorption kinetics are the main issues that limiting the use of metal hydrides in hydrogen storage applications. Kinetics can be improved via use of either nanostructure or amorphous materials. The optimum hydrogen storage device should have excellent stability and cyclability. Some alloys suffer from hydrogen embrittlement. On the other hand, hydrogenation can induce structure change in different alloys. Ni–Pd–P alloys have extraordinary stability [28]. Adding B to these alloys increases the crystallization temperature and hardness [26,29,30,31,32]. Crystalline boron is stable and inert to acids. However, amorphous boron can respond strongly to acids and oxidize slowly at room temperature. Therefore, in Ni–Pd–P–B glassy alloys, boron can be easily dissolved in the acidic medium: for example, via electrochemical dealloying, leaving behind randomly distributed nanopores. In most alloys, dissolution of one component results in a change in structure or creation of new precipitation phases. These changes rarely occur in Ni–Pd–P alloys. Hence, the obtained nanoporous glassy alloys are expected to exhibit high corrosion resistance (improving cycle performances), excellent chemical and structural stability, high crystallization temperatures, and excellent hydrogen storage. The nanopores formed within the glassy alloy created rooms for physical and chemical hydrogen storage.

Electrochemical hydrogen storage is another method of storing hydrogen gas in a liquid or solid medium through use of an electric current [23,24,33]. This type of storage is often used in fuel cells, where hydrogen can be released and react with oxygen to create electricity. One advantage of electrochemical storage is that it is very efficient, as energy needed to split water molecules into hydrogen and oxygen is much lower than that needed to generate electricity from fossil fuels. Additionally, this method produces no harmful emissions, making it a clean and environmentally friendly option for power generation. Moreover, this technology has potential to help solve the world’s energy problems by providing a way to store energy for use when it is needed.

Another potential use for electrochemical hydrogen storage is storing energy from renewable sources, such as sunlight or wind. When renewable energy is generated, it is often not needed immediately, but if it could be stored via electrolysis, it could be used later when demand is higher. This would help to make renewable energy sources more viable and increase their contribution to overall energy.

However, several challenges are associated with electrochemical hydrogen storage, including the high cost of the equipment required and the difficulty of safely handling and storing large quantities of hydrogen gas. Additionally, this method is not yet widely used or well-understood, meaning that further research is needed before it can be adopted on a large scale.

In this work, we investigate the possibility of using an enhanced energy-storage device, such as a pseudocapacitor based on nanoporous metallic glass, as a suitable candidate system for electrochemical hydrogen storage.

## 2. Materials and Methods

An alloy ingot with an atomic composition of Ni_60_Pd_20_P_16_B_4_ was prepared via arc melting of highly pure elements in an Ar environment. A ribbon sample with a width of 2 mm and a thickness of 20 mm was produced via the melt-spinning technique on the prepared ingot. The structure of the ribbon sample was examined with x-ray diffraction using CuKα radiation.

Dealloying and corrosion tests were performed with a standard linear polarization (LP) procedure at a scanning rate of 50 mV/min. In the LP experiment, the metallic glass ribbon sample was used as the working electrode, with a mass load of about 0.04 mg, while Ag/AgCl and Pt were used as reference and counter electrodes, respectively. A dilute solution of H_2_SO_4_ (0.1 M) was used as the corrosive electrolyte.

All electrochemical tests were performed using three-electrode cells at room temperature and Autolab potentiostat–galvanostat system model PGSTAT302N (Metrohm AG, Herisau, Switzerland). After the electrochemical measurements were taken, the glass ribbon sample was washed with a mixed solution of distilled water and ethanol to remove any contamination and thus dried for scanning electron microscopy (SEM).

Cyclic voltammetry experiments were achieved at different scan rates, ranging from 25 to 150 mV/s. Galvanostatic charge–discharge measurements were conducted to hydrogen capacity *Q* through application of the following formulas [34]:

$$Q\;\left[mAh/g\right]=\frac{t_{D}\left[s\right]\;i\left[A\right]}{3.6\;m\left[g\right]}$$

where $t_{D}$, i, and m are the discharge time, current, and the active mass of the working electrode, respectively.

The charge–discharge experiments were performed at different constant currents (75, 100, 125, 150, and 200 mA/g) under a cutoff potential of 2.25 V vs. Ag/AgCl electrode in 1 M KOH solution. Characterization of porosity was performed with Nova 1200e surface area and pore analyzers (Quantachrome Ins., Boynton Beach, FL, USA). The adsorption–desorption isotherm was obtained through use of N_2_ gas at 77 K.

## 3. Results

**Figure 1:**
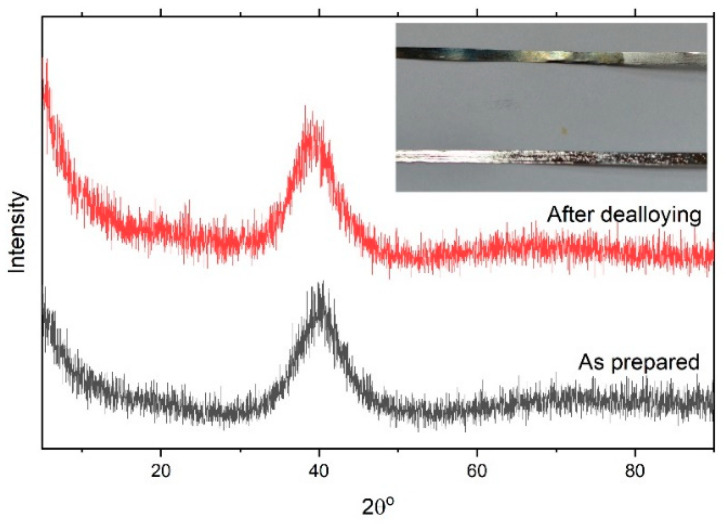
XRD pattern of the Ni_60_Pd_20_P_16_B_4_ glassy alloy ribbon sample. The inset shows photo images of the ribbon sample before and after the dealloying experiment.

**Figure 2:**
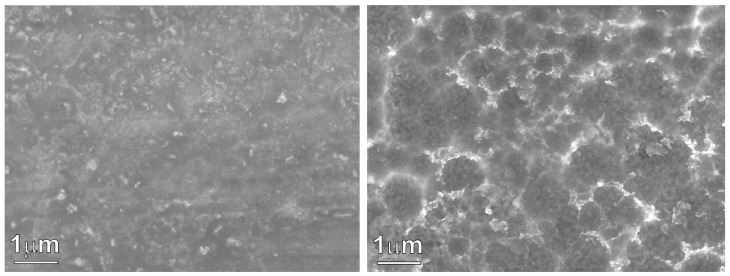
SEM images of the Ni_60_Pd_20_P_16_B_4_ glassy alloy ribbon sample before (**left**) and after electrochemical dealloying (**right**).

Figure 1 shows the XRD pattern of the as-prepared Ni_60_Pd_20_P_16_B_4_ glassy alloy ribbon. The alloy obviously had a typical amorphous metallic glass structure in which the broad peak was centered at 40°. This figure also shows the XRD pattern of the metallic glass ribbon after the chemical dealloying experiment. The alloy glass ribbon can be easily concluded to maintain its amorphous structure, indicating high structure stability. After the dealloying experiment, the amorphous structure did not change, even though the boron element had completely dissolved. This is because the amount of boron was small, so it was not enough to initiate crystallization; only surface deformation occurred as a result of disappearance of this element and nanopores were formed. In general, the Ni–Pd–P glassy alloy structure could be described as a randomly filled dense structure with short-range chemical order. The excellent stability of these glassy alloys mainly arises from the instability of the corresponding individual binary phases instead of the atomic arrangements in these alloys [35]. Indeed, stable amorphous materials that show no structural transformation upon chemical treatment are favorable for reversible hydrogen storage., especially in the case of pseudocapacitors, where the mechanism of storage is mainly faradaic. Pseudocapacitors suffer from low life cycle and low stability. Through incorporation of a glassy alloy into the structure of the pseudocapacitor, these two drawbacks can be overcome. The inset figure presents a photo image of glassy alloy ribbon samples before and after chemical dealloying. The affected area shows a change in color. This color change indicates the success of the dealloying process. Figure 2 shows an SEM image of a glassy alloy sample before and after dealloying. The smooth and homogeneous nature of the glassy surface completely disappeared. Here, B was entirely dissolved, and the alloy composition changed from Ni_60_Pd_20_P_16_B_4_ to Ni_58_Pd_24_P_18_. Table 1 compares nomination and actual composition of the Ni_60_Pd_20_P_16_B_4_ alloy before and after electrochemical dealloying.

For decades, the phenomenon of dealloying as corrosion has been well known. Typically, dealloying appears in an alloy if at least two of the components have quite different equilibrium potentials in certain aggressive solutions (electrolytes). In these cases, when the alloy is polarized between these values, the reactive component is selectively dissolved, leaving behind a surface rich in residual, less-reactive elements. The unreacted metal surface acts as a passive-like film: that is, the result of a purely porous metallic film. This mechanism consistently creates layers, with diminutive fracture toughness, that have severely modified mechanical properties, causing many stress-corrosion cracking processes in alloys [36,37,38,39,40]. Dealloying was first established to deliver porous nickel, which is used as a catalyst in several chemical activities [41,42,43,44].

Selective dissolution or more-specified controlled dealloying is used in many commercially important alloys to produce tailored nanoporous metals for chemical-sensing and catalytic applications [45,46,47,48]. Size of pores can be tailored during formation of nanoporosity via modification of the electrolyte [49,50,51]. This coarsening process can be interpreted as an increase in surface mobility of the less-reactive atoms within the alloy. Collective activity of dissolution of the active elements from the top layer with surface diffusion of the less-reactive elements generates a porous nanostructure [52,53]. Many nanoporous alloys with tailored nanostructures can thus be prepared through controlled electrochemical dealloying. However, improvement is necessary to prevent porosity from coarsening during real-time application.

The linear polarization resistance (LPR) method, used widely in electrochemistry, comprises applying to the sample very small voltage differences (normally less than ±30 mV) relative to the sample’s open circuit potential (E_oc_) to monitor corrosion behavior in industrial applications. When DC voltage is changed slightly, a current is induced to flow between the working electrodes and the counter. Over a narrow range around corrosion potential, the current response is linear. Therefore, the material’s resistance to polarization, defined as polarization resistance (*Rp*), can be obtained from the slope of the linear part of the polarization curve (Tafel’s plot). This polarization resistance is basically inversely proportional to corrosion current density ($j_{cor}$), where larger *Rp* values indicate better corrosion resistance, as shown by the following formula [54]:

$$j_{cor}=\beta {\left[\frac{\Delta j}{\Delta E}\right]}_{\Delta E\to 0}$$

where *E* is potential (V), $j_{cor}$ is corrosion current density (A/cm^2^), and β is the constant associated with anodic $\left(\beta_{a}\right)$ and cathodic $\;(\beta_{c})$ (Tafel slopes), so that:

$$\beta =\frac{\beta_{a}\;\beta_{c}}{2.3\;\left(\beta_{a}+\beta_{c}\right)}$$

**Figure 3:**
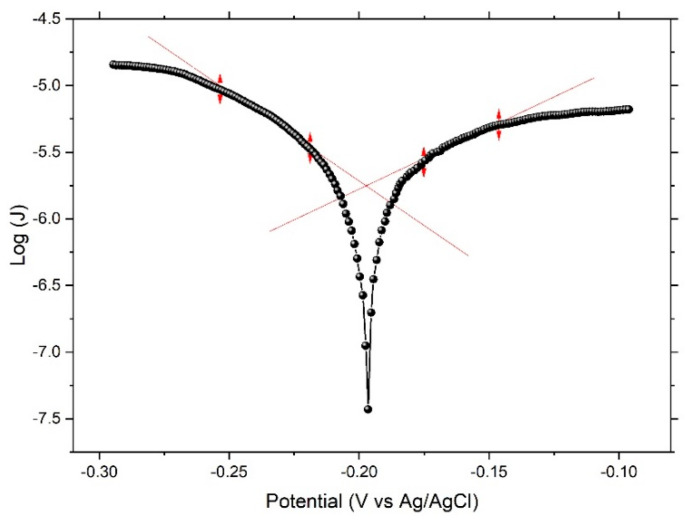
Linear polarization curve (Tafel’s curve) for the Ni_60_Pd_20_P_16_B_4_ glassy alloy ribbon sample in 0.1 M H_2_SO_4_ solution.

Figure 3 displays a typical current–potential curve (Tafel’s polarization curve) of electrochemical dealloying of the Ni_60_Pd_20_P_16_B_4_ glassy alloy ribbon in 0.1 M H_2_SO_4_, with a scanning rate of 50 mV/min. The shape of this curve indicates success of the dealloying process. Here, B was the most reactive element in the sample. When applied potential was fairly higher than equilibrium potential (*E*_c_) of B, a low current hill implied surface passivation via a thin, less-reactive residual-metal layer. Only at *E*_c_ did the current rise significantly and the boron continue to dissolve generating three-dimensional roughness, hence a nanoporous structure was formed. Despite the amount of boron being very small inside the alloy, when it was completely dissolved, homogeneity of the surface was greatly affected. Distributed nanopores were also formed, and their density was detected using adsorption isotherm measurements. All important deduced parameters are tabulated in Table 2.

**Figure 4:**
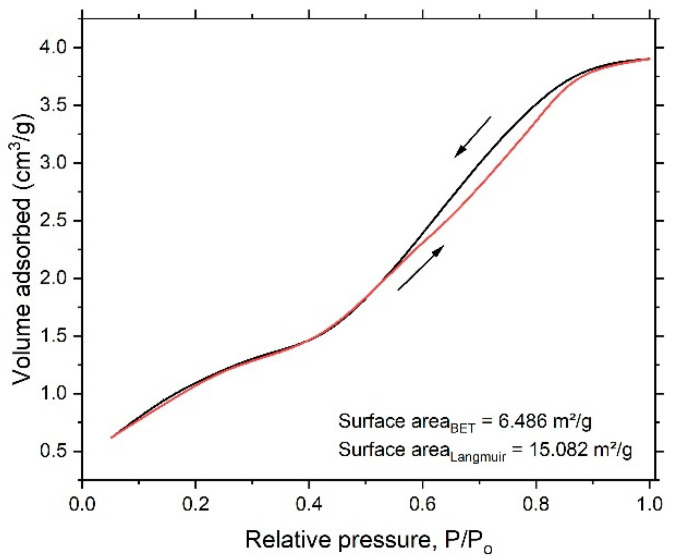
N_2_ adsorption/desorption isotherm for the dealloyed sample.

**Figure 5:**
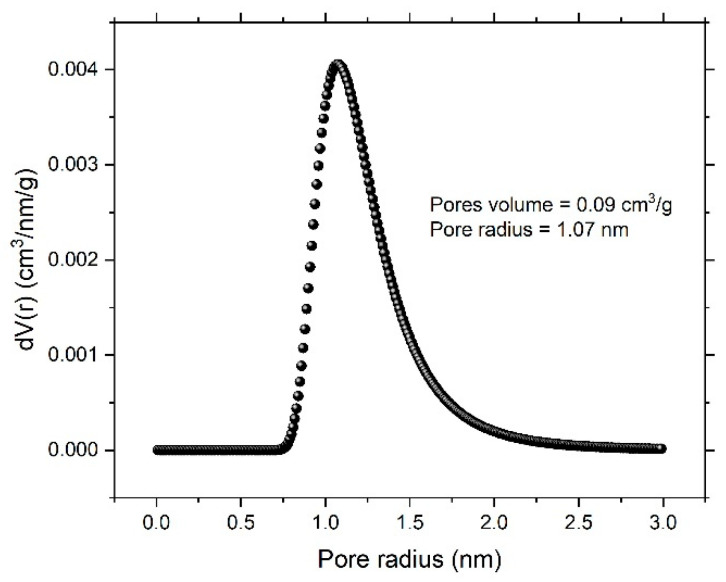
Pore-size distribution curve obtained according to the Dubinin–Astakhov (DA) method.

To account for specific surface area and the formed nanoporous structure, a N_2_ adsorption/desorption experiment was performed. Figure 4 shows the N_2_ adsorption/desorption isotherm for the dealloyed sample. The obtained isotherm displayed an IV-type model, indicating the size of the formed pore within a few nanometers. The Brunauer–Emmett–Teller (BET) and Langmuir methods were performed to obtain total surface area of the dealloyed sample. The obtained values were 6.486 m²/g and 15.082 m²/g, respectively. The Dubinin–Astakhov (DA) method, which provides data on overall pore distribution, was used in analysis of the adsorption isotherm to obtain the pore radius. Figure 5 shows the pore-size distribution curve, showing a sharp peak centered at 1.07 nm and indicating predominance of mesopores; the calculated nanoporous volume was found to be around 0.09 cm^3^/g.

**Figure 6:**
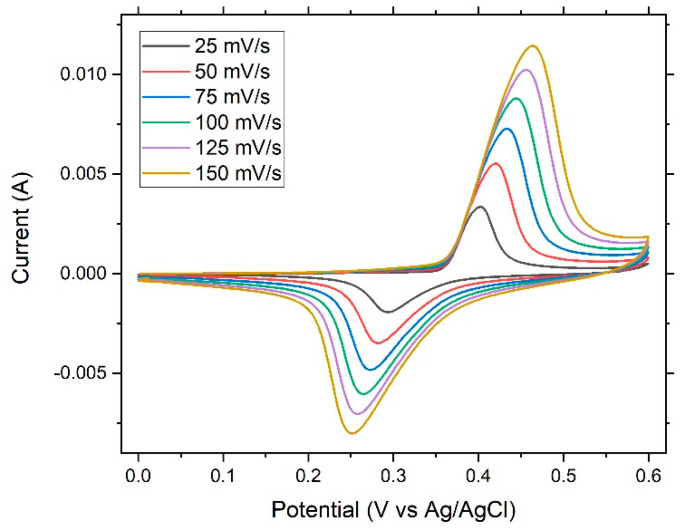
Cyclic voltammetry curves of the dealloyed glassy alloy ribbon sample at different scanning rates, as indicated.

**Figure 7:**
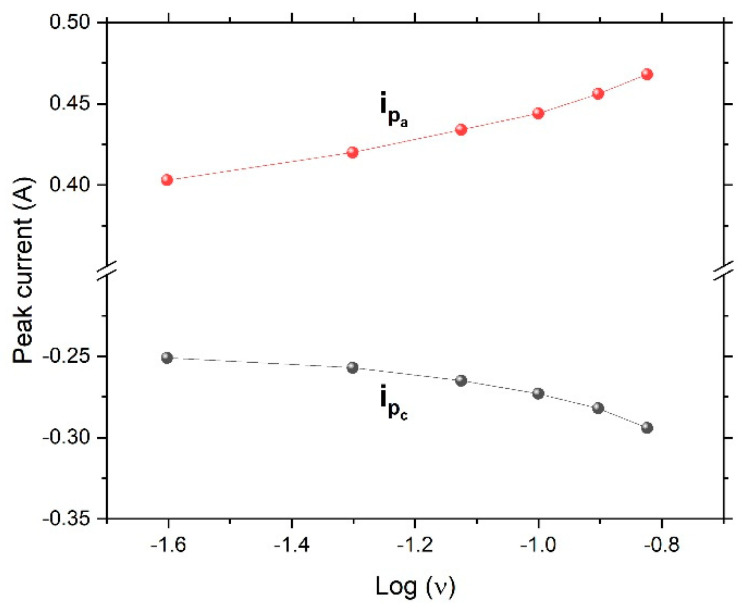
Trumpet-shaped plot of peak current versus the logarithm of the scan rate, ν (V/s), indicating reversible (low-barrier) electron-transfer kinetics (i_pa_ and i_pc_ are the anodic and cathodic peak currents, respectively).

Figure 6 shows the cyclic voltammetry curves of the dealloyed ribbon sample. The figure revealed an ideal shape for pseudocapacitors. Oxidation and reduction peaks were present. With an increased scanning rate, the oxidation peaks shifted to the right, while the reduction peaks shifted to the left, giving rise to a trumpet plot (Figure 7) and suggesting reversible (low-barrier) electron-transfer-reaction kinetics between the electrode and the electrolyte; thus the electrochemical kinetics could be determined via fitting to the Butler–Volmer or Marcus model.

Energy storage in pseudocapacitors can be executed through faradaic reactions. The pseudocapacitors store charges electrostatically; transfer of charge can be executed between electrode and electrolyte. Once voltage is applied to a pseudocapacitor, both reduction and oxidation occur on the material of the electrode. The faradaic process used in these capacitors enhances electrochemical reactions, providing greater specific capacitance and energy densities compared to other supercapacitors. In fact, owing to the amorphous nature of metallic glass, highly reversible effects that lead to better cycle stability due to the absence of a phase transition are significantly expected. Pseudocapacitors have higher specific capacitances, higher energy density, and lower-cycle life or stability compared to supercapacitors. Use of metallic glass instead of metal oxides or conducting polymers to manufacture pseudocapacitors will overcome the problems of low stability and improved energy storage.

**Figure 8:**
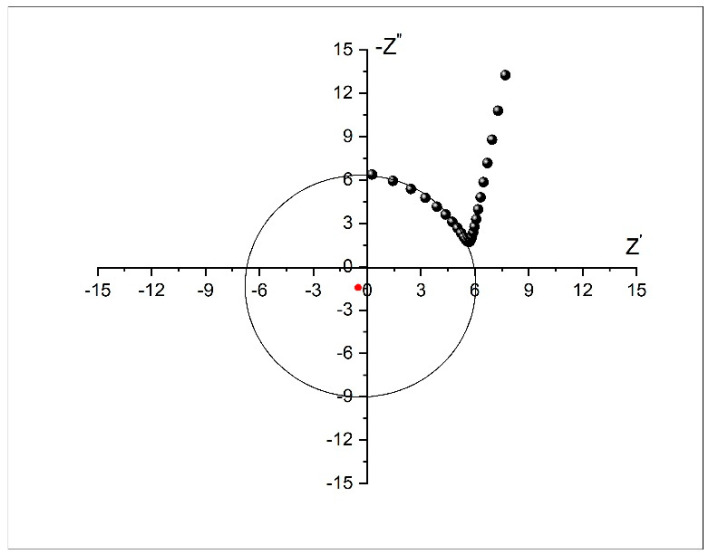
Cole–Cole (Nyquist) plot of the dealloyed glassy alloy ribbon sample.

Impedance of the dealloying sample was measured at different frequencies through use of electrochemical impedance spectroscopy (EIS). Figure 8 shows the Cole–Cole (Nyquist) plot measured from 100 kHz to 100 Hz. The plot establishes a semicircle with a radius of ~6 Ω at high frequency, suggesting low interfacial charge-transfer resistance (Rct), and an approximately vertical Warburg slope at low frequency, suggesting fast diffusion of ions to the electrode surface.

**Figure 9:**
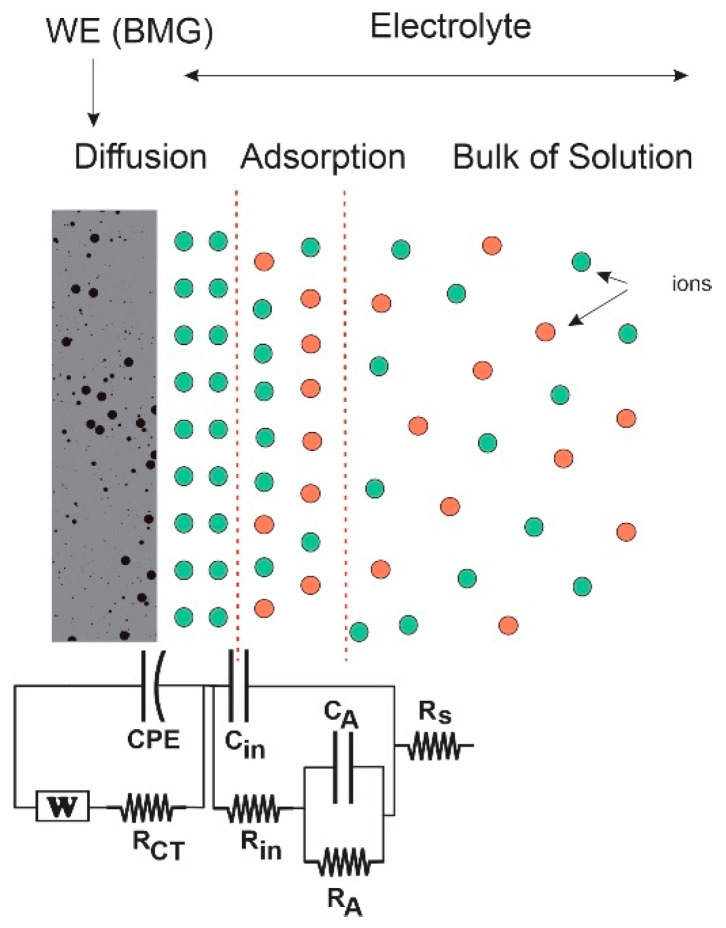
Equivalent circuit with the Warburg and constant phase (CPE) elements used in this work (red circles denote negative ions while green cycles denote positive ions).

Three points are noted here. Appearance of negative resistance has been observed experimentally in several systems [55,56,57]. This negative value arises due to the process of active-to-passive transition on the surface of the nanoporous metallic glassy alloy. Secondly, the center of the semicircle lies below the X-axis. In most real systems, the Nyquist plot is likely to be a semicircle with the center on the X-axis. However, the observed plot here is indeed a semicircle, but the center is located below the X-axis. This depressed semicircle could have appeared due to high surface roughness of the glassy alloy sample. The porous nature could have led to the inhomogeneous reaction rates on the surface due to distribution of active sites (with varying activation energies) on that surface. Another possible explanation depends on the process of the dealloying experiment. The dealloying process resulted in formation of a higher concentration of pores on the surface and fewer within the bulk of the materials. This pore-concentration regression led to conductivity variation along the path from the top surface to the inside of the material [58,59]. Generally, it can be said that in some cases, especially for metals, a depressed semicircle will appear due to surface inhomogeneity, surface roughness, electrode porosity, surface disorder, or geometric irregularities of the working electrode. In this case, the pure capacitor should be replaced by a constant phase element (CPE) to compensate for depression of the semicircle, as shown in Figure 9.

For modeling semi-infinite linear diffusion in a large planar electrode, Warburg impedance is the most common circuit element. The Warburg equation can be expressed as:

$$Z_{W}=\sigma \omega^{-1/2}-j\sigma \omega^{-1/2}$$

where σ is the Warburg coefficient, with units of $\Omega \;s^{-1/2},$ and can be extracted from measurement data and calculated according to the following:

$$\sigma =\frac{RT}{n^{2}F^{2}A\sqrt{2}}\left(\frac{1}{c_{O}^{b}D_{O}^{1/2}}+\frac{1}{c_{R}^{b}D_{R}^{1/2}}\right)$$

where *R* and *F* are the gas and Faraday constants, *D* is the diffusion coefficient and c is the concentration of elements in the bulk. The indices *O* and *R* denote the oxidized and reduced elements, respectively.

**Figure 10:**
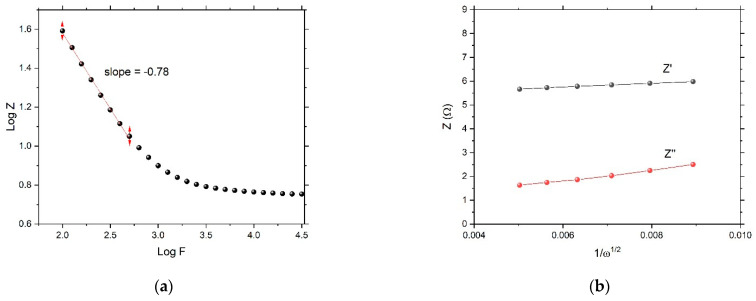
(**a**) Warburg Bode plot and (**b**) Warburg impedance plot.

In the Nyquist plot, the Warburg impedance, associated with restricted, controlled diffusion, is extremely noticeable under EIS as a straight line with a phase of 45°. In Figure 8, the Warburg vertical slope is not 45°. Figure 10a shows the Warburg plot. At the lower frequencies where Warburg impedance leads, the slope of |Z| should have been −0.5 (different from the pure capacitor, which had a slope equal to −1). Here, the calculated slope was −0.78. When charge-transfer resistance and double layer capacitance are present, they hide the Warburg impedance element attribute and become difficult to identify. At higher frequencies, charge-transfer resistance leads, and the phase angle becomes 0°. Figure 10b is characteristic to Warburg impedance. Both the real and imaginary parts of Z are plotted vs. $1/\omega^{2}$. The lines are straight and parallel, and the slope is equal to Warburg’s constant, σ. The intersection of real impedance with the Z-axis is R_CT_, while the imaginary impedance line must intersect with the Z-axis at the origin. These two parallel lines are usually used to identify the Warburg element.

Pseudocapacitors store charges by transferring them between the electrode and the electrolyte. The resultant capacitance (pseudo) is proportional to the charge transferred in this process and can be expressed as follows:

$$C_{pc}=q\frac{dS}{dV}$$

where *q* is the charge required for (faradaic) adsorption/desorption of ions, dS is exposure area of the surface and *dV* is change in potential [60]. Thus, according to this equation, capacitance is seldom constant; therefore, it is called pseudocapacitance.

Due to the faradaic process, pseudocapacitors attain much higher energy density and specific capacitance than can other storage devices. For a metallic glass sample, the nature of the faradaic process can be explained with a redox (pseudo) capacitance mechanism and/or an intercalation (pseudo) capacitance mechanism. The redox reaction is achieved via electrochemical adsorption of ions on the surface at redox active sites (surface-charge storage). Examples of pseudocapacitive materials include metal-based compounds and conducting polymers. Intercalation (pseudo) capacitance is based on diffusion of charged ions into the layer without crystal structural transformation. Vanadium pentoxide and niobium oxide are the most studied pseudocapacitive materials for this type of storage mechanism. To obtain information about kinetic behavior of electrodes, it is crucial to distinguish between the capacitive and diffusion contributions of pseudocapacitive material in an electrochemical activity.

**Figure 11:**
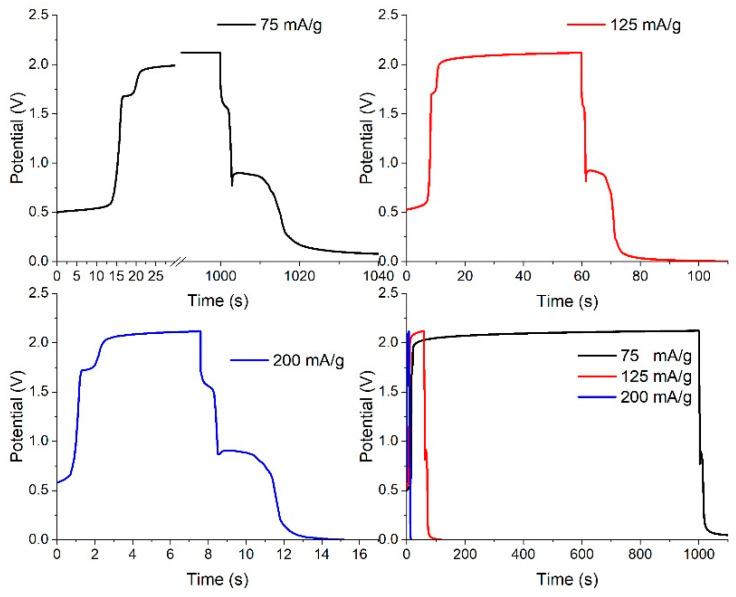
Charge–discharge curves of a dealloyed glassy alloy ribbon sample at different constant currents.

Galvanostatic charge–discharge curves can also describe pseudocapacitive performance. In them, pseudocapacitive activity without any crystal structural transformation is identified by a linear shape of potential vs. capacity. Figure 11 shows the charge–discharge curves of a dealloyed metallic glassy ribbon sample.

**Figure 12:**
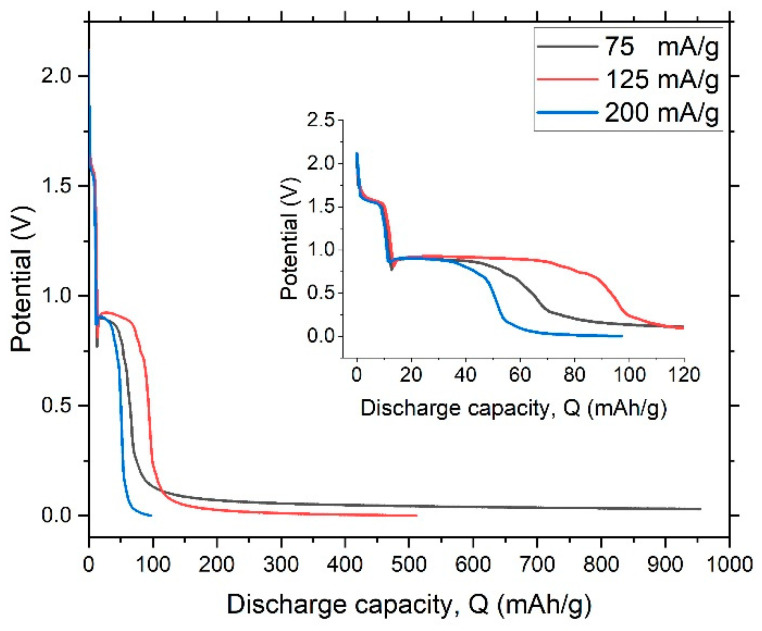
Discharge-potential curve of the dealloyed glassy alloy ribbon sample at different constant currents.

Figure 12 shows the discharge-potential curve of the first cycle at a constant current (0.0009 A). The process of discharging clearly took place in three stages. Despite difference in intensity of the current, the shapes of the charge and discharge curves are very similar, indicating a high degree of stability. The multiple chemical processes involved in charge–discharge suggested that the dealloyed glassy metal could be modeled as a circuit of three capacitors, as illustrated in Figure 9. Discharge behavior is the vital factor that controls reliability of output power in storage devices. The plateau in discharge potential determines the discharge characteristic of the dealloyed sample, as shown in Figure 12. The longer and flatter the plateau, the better the discharge performance.

The electrochemical charging–discharging reactions can be described by the following equation:

$$M+H_{2}O+e^{-}↔\mathrm{MH}+{\mathrm{OH}}^{-}$$

where M is the metallic glassy alloy.

There are two possible mechanisms for H storage in a prepared nanoporous glassy alloy: (a) The hydrogen atoms from the electrolyte gradually diffuse through the adsorbed layer formed on the rough surface and then through alloy–electrolyte interface into the bulk of the material to form metal hydrides. The presence of Pd enhances hydrogen diffusion due to its hydrogen-catalytic features. (b) The hydrogen atoms travel through pores and form hydrogen molecules that can be stored physically within available empty volume, whereas during the discharging process, H diffuses from the bulk alloy to the surface and then reacts with oxygen to generate a water molecule.

The diffusion rate of H atoms within the surface layer of the alloy is a major factor that affects charging-current utilization. The positive effect of the presence of pores helps enhance hydrogen diffusion rates and charge transport via increasing interface area and creating volumes for physical hydrogen storage. The amorphous structure facilitates uniform distribution of pores and thus enlarges the reactive sites for metal hydride formation [61,62,63].

To account for high-rate dischargeability (HRD), the working electrode was discharged at different constant currents (A = 75, 100, 125, 150, and 200 mA/g). HDR is defined as:

$$\mathrm{HRD}\;\left(\%\right)=\left(C_{A}/C_{75}\right)\times 100$$

where *C_A_* is the maximum capacity at the selected current density.

**Figure 13:**
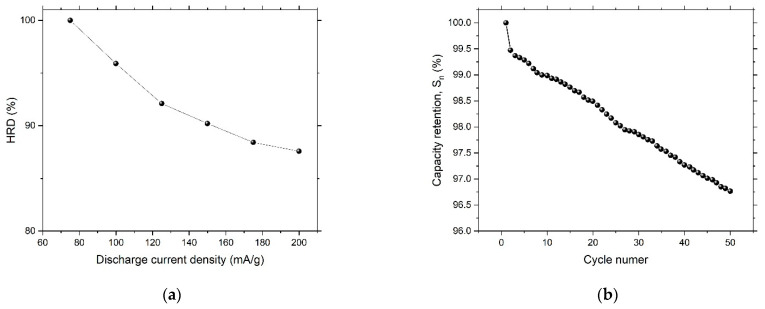
(**a**) High-rate dischargeability (HRD) and (**b**) capacity retention ($S_{n}$) for the dealloyed glassy alloy ribbon sample.

To examine cycling durability of the dealloyed electrode, capacity retention ($S_{n}$) is described by the following relation:

$$S_{n}\left(\%\right)=\left(C_{n}/C_{max}\right)\times 100$$

where $S_{n}$ is capacity retention at *n* cycles, $C_{n}$ is discharge capacity at *n* cycles and $C_{max}$ is the maximum discharge capacity. The calculated capacity retention rate at the 40th cycle was 97%, which reveals high stability. Figure 13 shows high-rate dischargeability (HRD) and capacity retention $S_{n}$ for the dealloyed glass alloy sample.

## 4. Conclusions

In this work, a glassy alloy ribbon sample with an atomic composition of Ni_60_Pd_20_P_16_B_4_ was prepared via arc melting of pure elements in an Ar environment. According to XRD analysis, the alloy had a typical amorphous metallic glass structure, in which the broad peak was centered at 40°. After chemical treatment, the ribbon sample maintained this amorphous structure, indicating high structure stability that is highly desirable for reversible hydrogen storage. After electrochemical dealloying in 1 M H_2_SO_4_, the smooth and homogeneous nature of the glassy surface completely disappeared, and B was entirely dissolved. Thus, the alloy composition changed from Ni_60_Pd_20_P_16_B_4_ to Ni_58_Pd_24_P_18_. Adsorption isotherms were used to calculate specific surface area, pore radius, and pore volume. The obtained values were 15.082 m^2^/g, 1.07 nm, and 0.09 cm^3^/g, respectively. Cyclic voltammetry of the nanoporous ribbon sample revealed the pseudocapacitive nature of this alloy. Impedance measurement showed that both low interfacial charge-transfer resistance and fast diffusion of ions to the electrode surface are possible charge storage mechanisms. Charge–discharge experiments performed in 1 M KOH showed that the dealloyed sample had a maximum storage capacity of approximately 950 mAh/g. High-rate dischargeability (HRD) and capacity retention ($S_{n}$) indicated high stability.

## Figures and Tables

**Table 1:** Nomination and actual composition of the Ni_60_Pd_20_P_16_B_4_ alloy before and after electrochemical dealloying.

Nominal Composition	Actual Composition							
wt. %	wt. %				at. %			
	Ni	Pd	P	B	Ni	Pd	P	B
Ni_60_Pd_20_P_16_B_4_	56.42	34.89	7.98	0.71	59.65	20.34	15.99	4.02
Ni_58_Pd_24_P_18_	52.38	38.73	8.89	−	57.81	23.59	18.6	−

**Table 2:** Summary of electrochemical parameters obtained from the polarization curve of the Ni_60_Pd_20_P_16_B_4_ glassy alloy ribbon sample.

	$\beta_{a}$ (mV)	$\beta_{c}$ (mV)	$E_{corr}$ (mV)	$J_{corr}$ (mV/m^2^)	Corrosion Rate (mm/Year)	$R_{P}$ (W)
Ni_60_Pd_20_P_16_B_4_	0.0627	0.0566	−0.196	1.57	0.010222	10291

## Data Availability

Not applicable.
